# Cyanophycin modifications for applications in tissue scaffolding

**DOI:** 10.1007/s00253-024-13088-4

**Published:** 2024-03-15

**Authors:** Natalia Kwiatos, Deniz Atila, Michał Puchalski, Vignesh Kumaravel, Alexander Steinbüchel

**Affiliations:** 1 International Centre for Research on Innovative Biobased Materials—International Research Agenda (ICRI-BioM), Lodz University of Technology, Stefanowskiego 2/22, Łódź, Poland; 2 Institute of Material Science of Textile and Polymer Composites, Lodz University of Technology, Żeromskiego 116, Łódź, Poland

**Keywords:** Multi-L-arginyl-poly-L-aspartate, Crosslinking, Glutaraldehyde, Genipin, EDC/NHS

## Abstract

**Abstract:**

Cyanophycin (CGP) is a polypeptide consisting of amino acids—aspartic acid in the backbone and arginine in the side chain. Owing to its resemblance to cell adhesive motifs in the body, it can be considered suitable for use in biomedical applications as a novel component to facilitate cell attachment and tissue regeneration. Although it has vast potential applications, starting with nutrition, through drug delivery and tissue engineering to the production of value-added chemicals and biomaterials, CGP has not been brought to the industry yet. To develop scaffolds using CGP powder produced by bacteria, its properties (*e.g.*, biocompatibility, morphology, biodegradability, and mechanical strength) should be tailored in terms of the requirements of the targeted tissue. Crosslinking commonly stands for a primary modification method for renovating biomaterial features to these extents. Herein, we aimed to crosslink CGP for the first time and present a comparative study of different methods of CGP crosslinking including chemical, physical, and enzymatic methods by utilizing glutaraldehyde (GTA), UV exposure, genipin, 1-ethyl-3-[3-dimethylaminopropyl] carbodiimide hydrochloride/N-hydroxysuccinimide (EDC/NHS), and monoamine oxidase (MAO). Crosslinking efficacy varied among the samples crosslinked via the different crosslinking methods. All crosslinked CGP were non-cytotoxic to L929 cells, except for the groups with higher GTA concentrations. We conclude that CGP is a promising candidate for scaffolding purposes to be used as part of a composite with other biomaterials to maintain the integrity of scaffolds. The initiative study demonstrated the unknown characteristics of crosslinked CGP, even though its feasibility for biomedical applications should be confirmed by further examinations.

**Key points:**

• *Cyanophycin was crosslinked by 5 different methods*

• *Crosslinked cyanophycin is non-cytotoxic to L929 cells*

• *Crosslinked cyanophycin is a promising new material for scaffolding purposes*

**Graphical Abstract:**

**Figure:**
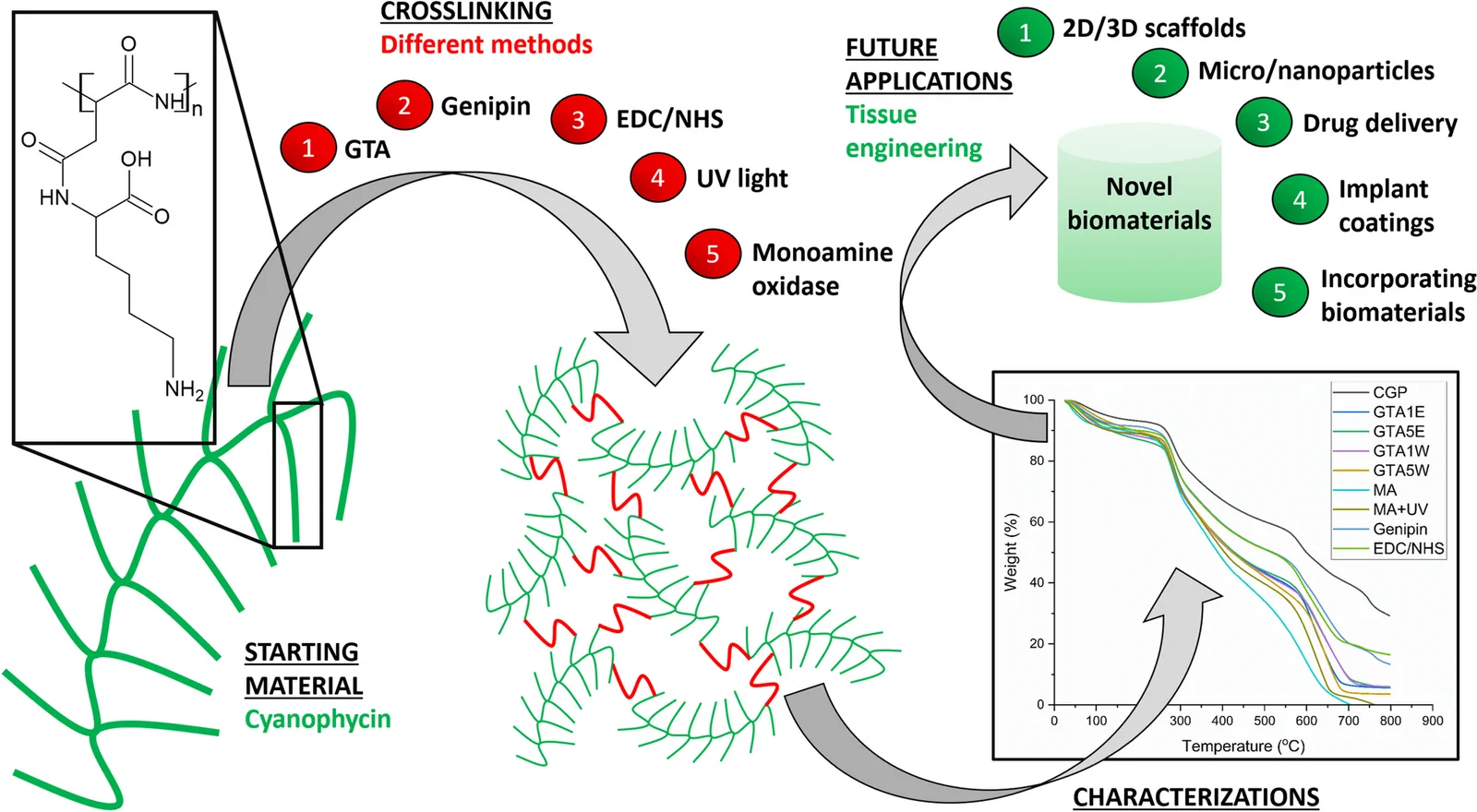

**Supplementary Information:**

The online version contains supplementary material available at 10.1007/s00253-024-13088-4.

## Introduction

Cyanophycin, *i.e.*, cyanophycin granule polypeptide (CGP) or multi-L-arginyl-poly-L-aspartate, is a specific type of material among a vast variety of natural polypeptides which consists of amino acids—namely (i) aspartic acid in the backbone and (ii) arginine in the side chain with a high polydispersity of molecular mass ranging within 8 and 100 kDa (Kwiatos and Steinbüchel 2021). Its chemical structure may differ depending on the cultivation conditions of CGP-synthesizing bacteria and the type of the strain. For instance, arginine units could be replaced by other amino acids—most commonly lysine (Frommeyer et al. 2016). This biopolymer, which belongs to the polyamides, is produced by cyanobacteria (*e.g.*, *Anabaena cylindrica*) as first discovered in history (Mackerras et al. 1990), and heterotrophic bacteria (*e.g.*, *Clostridium botulinum*) (Aravind et al. 2016) to store carbon, nitrogen, or/and energy, then to use it transiently as an extracellular source for growth. CGP is synthesized by the enzyme cyanophycin synthetase (CphA) and could be built of individual amino acids or dipeptides (Sharon et al. 2021, 2022).

CGP is composed of amino acids which are safe building blocks that could be used in form of peptides, among others, as cell-penetrating peptides (Grogg et al. 2018b; Yokoo et al. 2021), drug delivery systems (Davoodi and Shafiee 2022), antimicrobial peptides (Scavello et al. 2022), anticancer agents (Yan et al. 2022), and elements for tissue scaffolding (Serrano-Aroca et al. 2022). In this area, CGP was studied before to be used as cell-penetrating peptides (Grogg et al. 2018a; Kurth et al. 2018), inhibitors of tyrosinases^13^, and as part of wound dressings (Uddin et al. 2020). The biocompatibility of CGP was studied on Chinese Hamster Ovary cells. The films prepared by crosslinking CGP chains with glutaraldehyde (GTA) showed no toxicity and a higher growth rate of the cells when compared to control—polystyrene (Tseng et al. 2016). In the field of tissue engineering, scaffolds have been constructed by using biomaterials with tissue-specific characteristics. To this respect, CGP—a white-brownish powder can be considered a good candidate since it has mechanical rigidity (Khlystov et al. 2017) making it a favorable component for developing alternative scaffolds, especially for hard tissues such as bones. Moreover, it may be regarded as suitable for designing non-fouling surfaces for selective adhesion of cells in the body after implantation owing to its zwitterionic features (Sällström et al. 2020). Furthermore, effective drug delivery systems can be developed by using CGP since it contains arginine. Recently, it was demonstrated that arginine-modified nanoparticles facilitated cellular uptake of these nanoparticles across the cell membrane, which was loaded with an anticancer drug (Yan et al. 2019). Besides, the intrinsic anti-cancerogenic characteristic of arginine was reported (Jahani et al. 2017). Other forms of value-added chemicals and/or biomaterials can also be formulated by modifying CGP (Kwiatos and Steinbüchel 2021); for instance, by incorporating lysine into it. It was previously revealed that polylysine modifications resulted in the enhancement of cell-scaffold interaction (Yuan et al. 2018), which might also be achieved by the use of the lysine-containing CGP. In addition, CGP has been considered due to its modest resemblance to RGD motifs (arginylglycylaspartic acid motif, *i.e.*, tripeptides with the amino acid sequence of arginine-glycine-aspartic acid), which are well-known cell attachment sites in the body (Tjong and Lin 2019). The presence of RGD motifs in biomaterials is highly appreciated to increase scaffold performance. Thus, the use of CGP has the potential to generate a similar enhancer effect on cell adhesion.

Nonetheless, to our knowledge, CGP is neither involved throughout the mentioned biomedical applications nor used industrially yet, despite its numerous potential applications. Hence, subsequent modifications of the polymer towards the targeted field of concept may greatly contribute to the applicability of CGP. Once the properties of CGP are discovered elaboratively, various tissue-engineered constructs can be designed in different forms (2-dimensional (2D) films, barrier membranes, patches, wound dressings, etc*.*, or 3-dimensional (3D) sponges, hydrogels, load-bearing discs, and non-load-bearing scaffolds, etc*.*). Furthermore, micro/nanoparticles containing CGP may serve as drug delivery platforms, reinforcing additives for mechanical performance, and models for a myriad of alternative applications. CGP or CGP-carrying starting solutions/materials can also be utilized to coat implant surfaces. Hence, cell-friendly surfaces can be created for the prevention of implant rejection in the body and stimulation of cell attachment, proliferation, spreading, and differentiation. CGP-containing scaffolds can be produced by using other biomaterials (polymers, bioceramics, etc*.*) which are incorporated with CGP or modified CGP to generate various functions such as antimicrobial or antioxidant actions. The synergistic effects of the combinational use of CGP (or modified forms of it) and other biomaterials can be analyzed deeply concerning the targeted field of application. To produce stable CGP structures (or to achieve a successful CGP incorporation into other structures), crosslinking can be considered a good strategy since it has been widely preferred to prepare scaffolds.

**Fig. 1:**
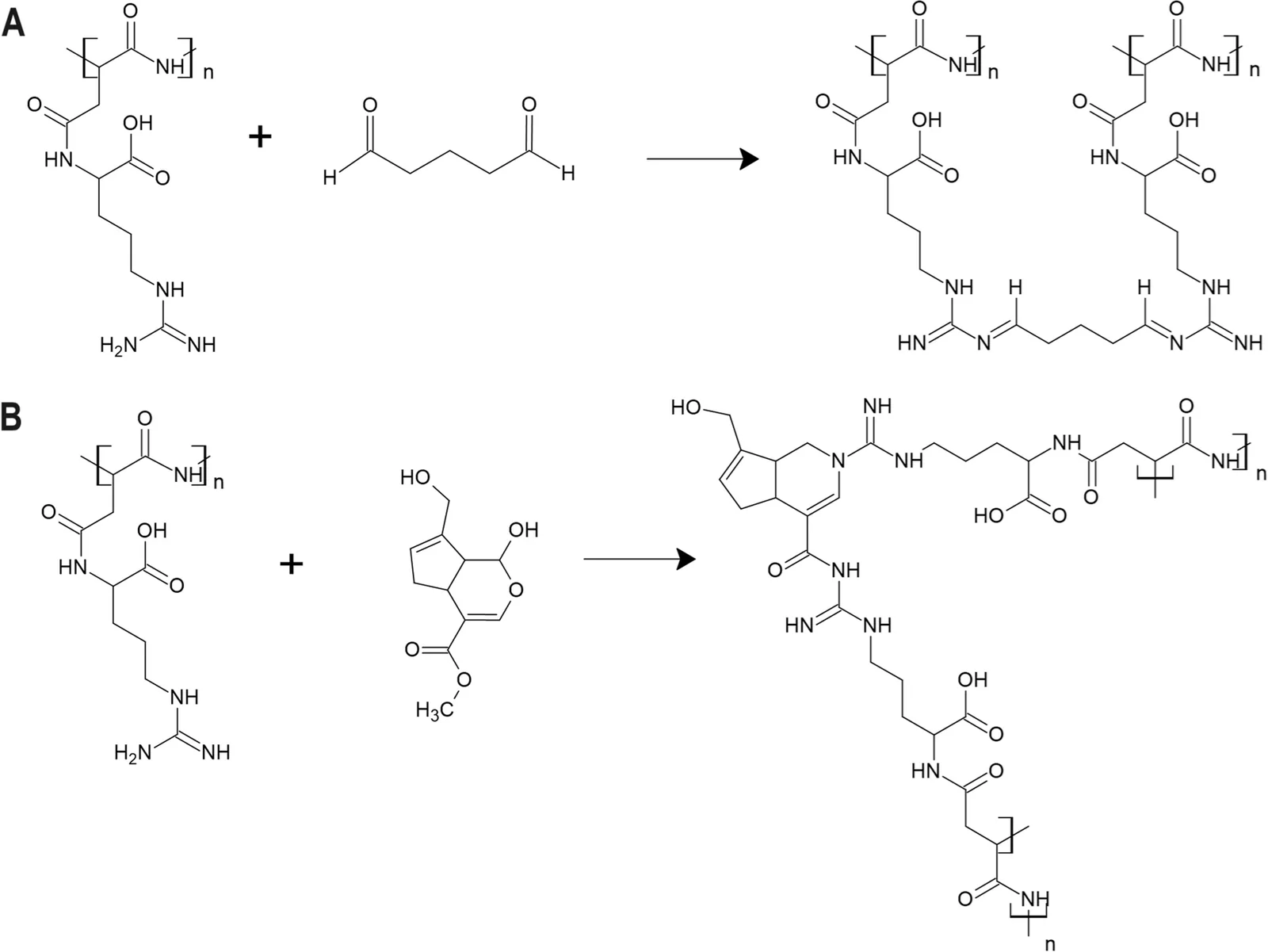

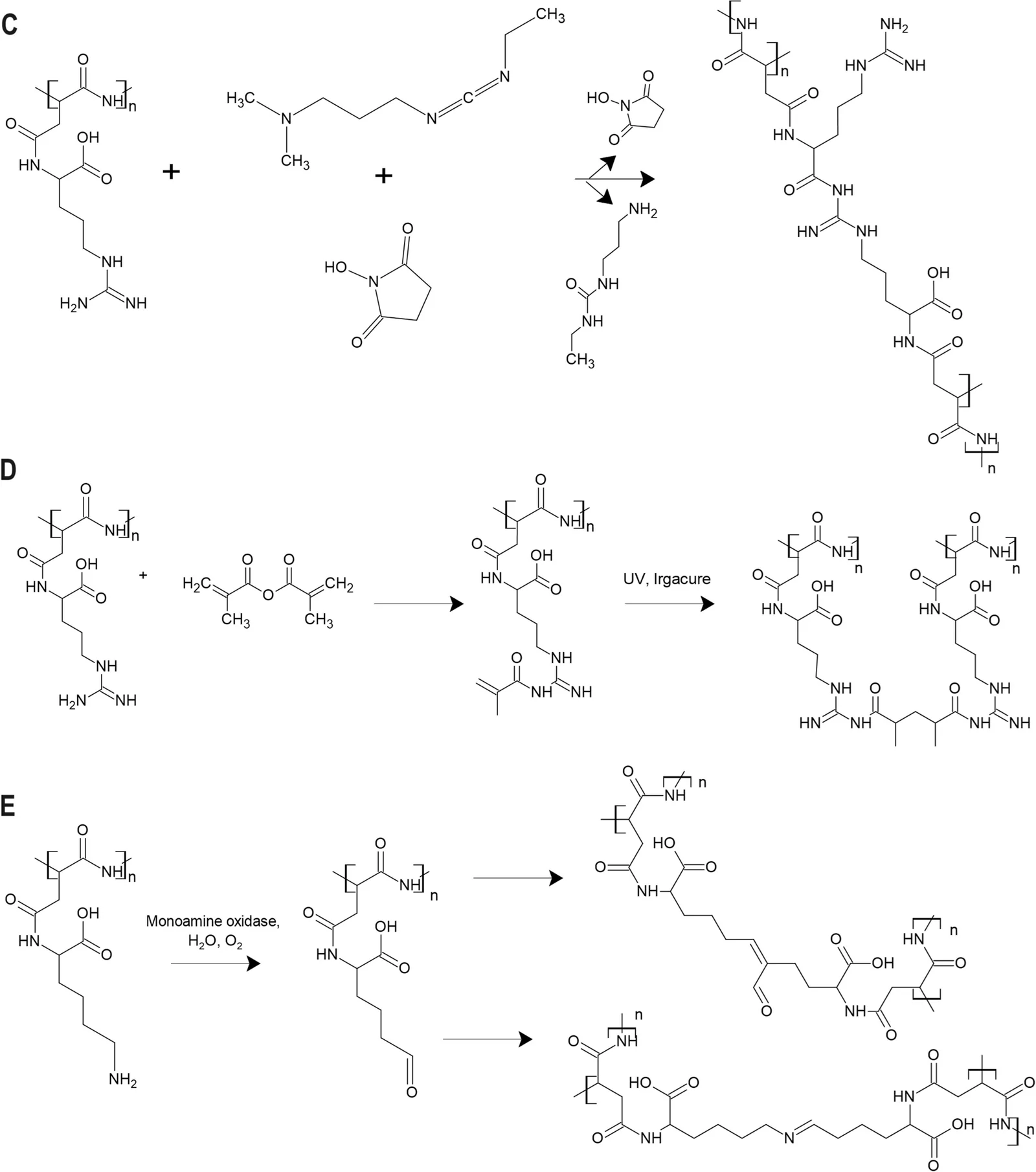
Presentations of the crosslinking reactions of CGP by utilizing the following crosslinkers: GTA (**A**), genipin (**B**), EDC/NHS (**C**), UV light (**D**), and MAO (**E**)

In this study, we aimed to investigate the influence of various crosslinking methods on CGP, including (i) chemical crosslinking by using crosslinker molecules: GTA (Fig. 1A), genipin (Fig. 1B), and EDC/NHS (Fig. 1C), (ii) physical crosslinking by UV exposure (photocrosslinking) after methacrylation (MA) of CGP (Fig. 1D), and (iii) enzymatic crosslinking by MAO (Fig. 1E). During the characterization studies, Fourier transform infrared spectroscopy (FTIR), differential scanning calorimetry (DSC), thermogravimetric analysis (TGA), solid-state proton nuclear magnetic resonance spectroscopy (^1^H NMR), and scanning electron microscopy (SEM) techniques were performed to analyze the properties of the crosslinked CGP samples. After the material characterizations, the CGP samples were analyzed in vitro using an L929 cell line to evaluate the effect of the different crosslinking methods on the cytotoxicity of the materials.

## Materials and methods

### Materials

CGP produced by *Escherichia coli* recombinant strain was kindly provided by Cysal GmbH (Münster, Germany). GTA 25% solution was purchased from Chempur, 1-(3-Dimethyl-aminopropyl)-3-ethylcarbodiimide from Thermo Scientific, and N-hydroxysuccinimide from TCI. The other chemicals were obtained from Merck and Chempur. All the chemicals had at least 98% purity. Human MAO was purchased from Sigma. All the chemicals used were of analytical grade and used as received without further purification.

### Methods

#### Crosslinking methods

##### Crosslinking with GTA

CGP powder (125 mg) was placed in each well of a 24-well plate. Next, the solution of 0%, 1%, and 5% of GTA in water or 70% in ethanol was prepared, and the powder was immersed in 1 ml of solution. Each condition was repeated in three wells. After 24 h of incubation at RT, the supernatant was removed, and the polymer was washed first with 0.2 M glycine (30 min, RT) and then with distilled water. Next, the samples were freeze-dried (Christ, Alpha 1–2 LD plus) at − 80 °C for 48 h.

##### Crosslinking with genipin

One hundred twenty-five milligrams of CGP powder was placed in each well of a 24-well plate. Next, the powder was immersed in 0% and 5% solution of genipin (40% ethanol in PBS). Each condition was done in triplicate. After 24 h, the samples were washed as explained above and freeze-dried at − 80 °C for 48 h.

##### Crosslinking with EDC/NHS

One hundred twenty-five milligrams of CGP powder was placed in each well of a 24-well plate. Next, the mixture of EDC and NHS was prepared in the ratio composition of 3:1, with EDC concentrations of 0 mM, 10 mM, 30 mM, and 60 mM. Each condition was repeated in triplicates. After 24 h of incubation at RT, the samples were frozen and lyophilized at − 80 °C for 48 h.

##### Crosslinking with UV exposure after methacrylation

One gram of CGP was weighed, placed in a beaker, immersed in 10 mL PBS, and then preheated to 60 °C. Next, 20% (v/v) of methacrylic anhydride was added to the mixture at the rate of 0.5 mL/min for 1 h with gentle stirring at 50 °C. To stop the reaction, the mixture was diluted 5 times with PBS at 40 °C. Next, the reaction mixture was dialyzed against distilled water using dialysis tubes with a cut-off of 12 kDa, for 1 week. Next, the polymer was filtered and air-dried. Next, it was immersed in 0.5% solution of Irgacure and subjected to UV irradiation (365 nm, 0.120 J/cm^2^) in a closed dark box for 1 h.

##### Crosslinking with MAO

Fifty microliters of a 1 mg/mL solution of purified CGP (soluble fraction prepared as explained in the previously described method (Wiefel and Steinbüchela 2014) was mixed with 150 µL potassium phosphate buffer (pH 7.4) and 50 µL of human MAO A of 68 U/mg (Sigma-Aldrich, M7316). The reaction was conducted with gentle shaking at 37 °C for 24 h. Next, the mixture was centrifuged, and the supernatant and precipitate were separated and lyophilized at − 80 °C for 48 h.

### Analytical methods

#### Fourier transform infrared spectroscopy (FT-IR)

The lyophilized samples of crosslinked CGP were subjected to analysis to verify crosslinking. FTIR was assessed using FTIR Nicolet 6700 spectrophotometer in the range of 400–4000 cm^−1^. The spectra of all samples were corrected by the background read of the atmosphere inside the FTIR spectrometer.

#### Differential scanning calorimetry (DSC)

The samples were evaluated for their thermal properties to detect any change after the modifications. DSC was performed with a heating rate of 5 °C/min by cycling from − 20 to 200 °C under an air atmosphere (DSC 2 STAR System, Mettler Toledo).

#### Thermogravimetric analyses (TGA)

The thermal stability of the samples was tested to elaborate on the effect of crosslinking methods on the modified samples. TGA curves of the samples were plotted via the Mettler Toledo TGA Star system with a heating rate of 10 °C/min in a temperature range of RT-750 °C in an air atmosphere.

#### Solid-state proton nuclear magnetic resonance spectroscopy (^1^H-NMR)

Solid-state ^1^H-NMR spectra of the control and the crosslinked groups were obtained with a Bruker AV III 600 spectrometer using a 2.5 mm probe and a sample rotation speed of 30 kHz under magic angle spinning (MAS).

#### Scanning electron microscopy (SEM)

Morphological features of the freeze-dried samples were observed utilizing scanning electron microscopy (Nova NanoSEM 230, FEI) to investigate the alterations in the sample morphology after the modifications. The observation was carried out under a low vacuum regime by the use of a secondary electron detector and beam energy of 10 keV.

### Cell culture methods

Mouse fibroblastic cell line—L929 (*i.e.*, mouse C3H/An connective tissue) was utilized for in vitro studies. The cells were seeded into 96-well plates with a cell density of 2 × 10^4^ cells/well and cultured in high glucose Dulbecco’s modified Eagle medium (DMEM) containing 10 (v/v) % FBS and 100 U/mL penicillin/streptomycin in a CO_2_ incubator at 37 °C. Meanwhile, gamma-sterilized samples (2 mg powder/100 µL media) were placed into the plates and incubated in the same culture media under the same conditions. The half volume of the media incubated with the samples was collected once in 2 days and replaced with fresh media. The half volume of the media in the cell-seeded plates was replaced with the collected media from the sample wells.

#### Cell viability

Cell viability was measured by the standard Alamar Blue assay at specific time points. After 24 h, 48 h, and 7 days of incubation, the culture medium was removed, and the cells were washed with sterile PBS. Then, Alamar Blue solution (DMEM/Alamar Blue reagent: 9/1) was added, and the plates were incubated at 37 °C in the dark for 4 h. After the incubation, the Alamar Blue solutions in the wells were transferred into a new plate to measure the absorbance at 570 and 600 nm. Cell-seeded tissue culture polystyrene (TCPS) well plates were set as the control group (100% viable).

#### Cell morphology

The cells were observed under an optical microscope at × 10 magnification to examine the changes in morphology after the indirect cytotoxicity tests conducted with the CGP samples.

#### Statistical analyses

One-way analysis of variance (ANOVA) was used to determine statistically significant differences between results during cell culture examinations. Tukey’s multiple comparison test for the post hoc pairwise comparisons (SPSS 22 Software Program, USA) was applied. Differences between groups were accepted as statistically significant at *p* < 0.05.

## Results

### Crosslinking methods applied and initial FTIR analysis

**Fig. 2:**
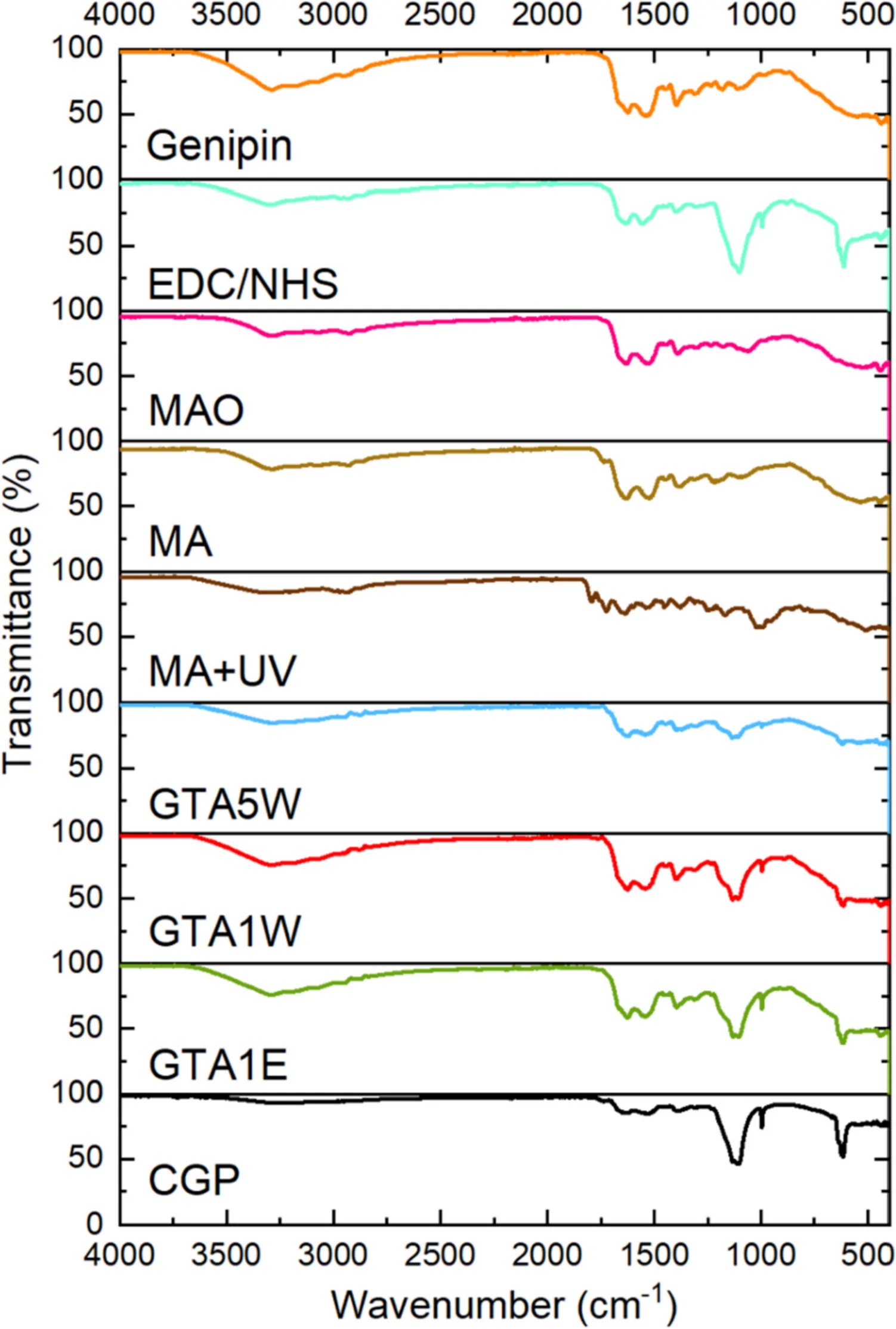
FTIR spectra of the samples: CGP, GTA1W, GTA5W, GTA1E, GTA5E, genipin, EDC/NHS, MA, MA + UV, and MAO

The crosslinking of CGP was performed independently by five different methods. The first presented method is crosslinking with GTA (Adamiak and Sionkowska 2020; Alavarse et al. 2022), which is one of the most commonly used chemical crosslinkers (Fig. 1). When covalent bonds are formed, the color of the material is changed to yellow, which could be observed in the case of CGP. In genipin crosslinking, lysine, and arginine of CGP can take part in the reaction (Fig. 1). As a result of the reaction with primary amines, deep dark pigments are formed (Bi et al. 2011; Adamiak and Sionkowska 2020). This was also confirmed by FTIR analysis, in which changes in transmittance in the range of 3000–3200 cm^−1^ and peaks at 1624 and 1541 cm^−1^ were observed, corresponding to the NH-stretching of primary amines (Fig. 2).

EDC/NHS Querycrosslinking allows the production of crosslinked molecules without any additional linker, by direct covalent binding between the molecules (Fig. 1). In the FT-IR spectra of crosslinked materials, only small shifts in peaks corresponding to NH-stretching are visible (Grabska-Zielińska et al. 2021; Rung et al. 2021). In the case of crude CGP and crosslinked CGP, the peak at 1541 cm^−1^ was shifted to 1560 cm^−1^ (Fig. 2).

The next method that was studied was the methacrylation of CGP and then crosslinking of the functionalized polymer by UV light (Fig. 1). Two peaks in 1724 and 1792 cm^−1^ corresponded to the formation of double bonds during methacrylation (Fig. 2). After UV irradiation (Fig. 2), these bonds were broken, CGP was crosslinked, and the peaks were no longer visible in the FTIR spectrum.

The enzymatic method with human MAO reaction was performed in physiological conditions as described in the methods section. As a result, a white precipitate was formed. FTIR spectrum indicated Schiff base formation (1640 cm^−1^) and change in primary amine composition (the new peak at 2922 cm^−1^) (Fig. 2).

### DSC

**Fig. 3:**
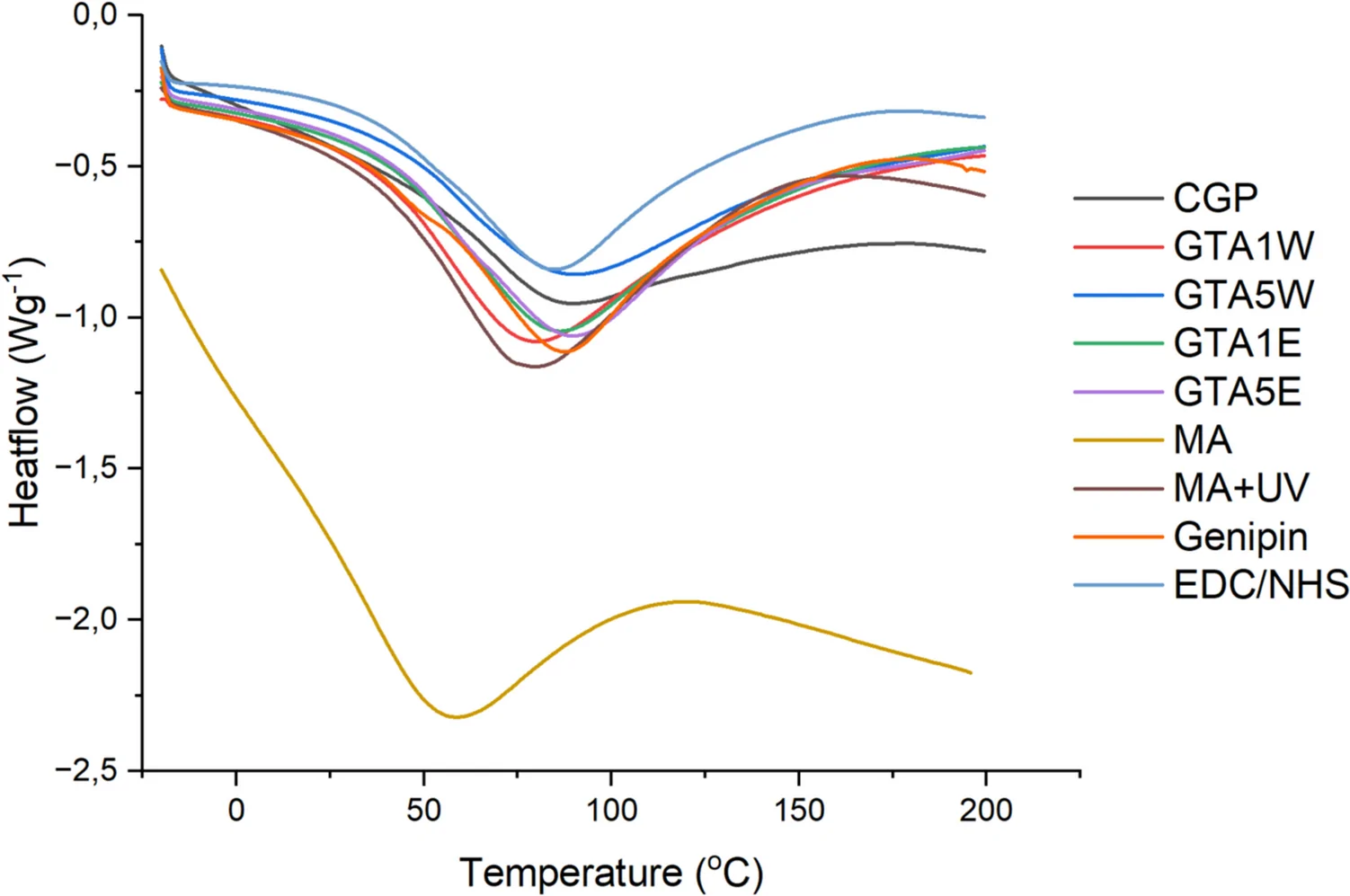
DSC thermograms of the samples before and after the modifications

The obtained samples were analyzed by DSC (Figs. 3 and S1) to measure the alterations in their thermal stability after the modifications. All samples displayed endothermic fall which could be divided into two stages concerning the change rate. The first stage observed from − 15 to 30 °C might be the representation of the glass transition temperature (Tg) of the samples where amorphous regions of the polypeptides responded to the temperature rise first and thus, become more mobile (Aldana et al. 2019). Among all the groups, crude CGP showed a faster fall with a higher slope of its curve, meaning that it might contain more amorphous parts compared to its crosslinked counterparts. The second stage of the endothermic fall beginning from 30 °C and ending at the peak levels was normally attributed to the denaturation of proteins (Mukherjee and Rosolen 2013). In the case of CGP, since it is not a protein, these peaks could be defined as the dissociation of aggregated peptides (Farrell et al. 2017).

### TGA

**Fig. 4:**
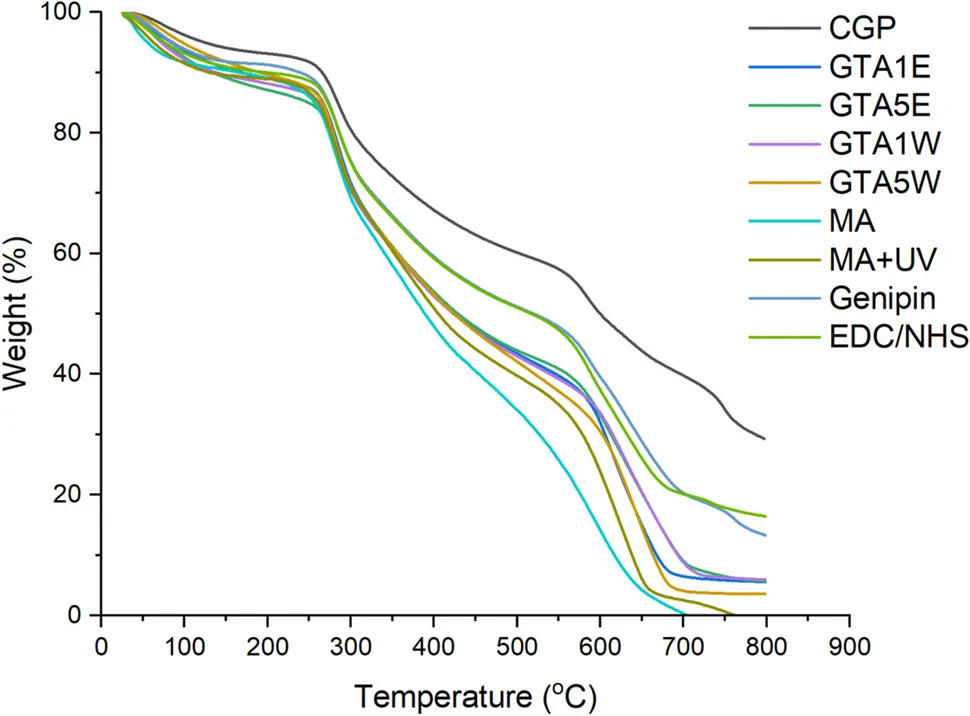
TGA curves of the samples before and after the modifications

Thermal degradation of the samples was studied by TGA analysis. CGP exhibits 5 stages of decomposition. The first weight loss occurred at about 100 °C, which indicates the presence of water remaining in the sample after lyophilization (Figs. 4 and S2). The polymer was stable up to around 200 °C. The major step of degradation started after 288 °C and contributed to a mass loss of around 50% (Table 1). As much as 30% of CGP remained after treatment at 800 °C. The temperature at the onset of decomposition was slightly increased for crosslinked CGP. The most rapid degradation was observed for the methacrylate samples, while the crosslinking with UV enhanced its thermal stability. The largest mass loss was observed for all samples in the temperature range of 280–600 °C (from 20% for crude CGP to 55% for MA + UV), and at this stage, the rate of degradation most significantly varied between the materials (Table 1).

**Table 1:** Temperature values correspond to the weight loss percentages of the samples obtained from TGA results

Sample/weight loss	10%	30%	50%	70%	95%
	Temperature (°C)				
CGP	265	365	597	785	-
GTA1W	137	300	422	614	800
GTA5W	190	302	422	601	684
GTA1E	166	302	428	603	800
GTA5E	132	300	428	612	800
MA	164	297	388	526	644
MA + UV	129	302	404	577	654
Genipin	238	324	521	643	-
EDC/NHS	198	320	513	630	-

### NMR

**Fig. 5:**
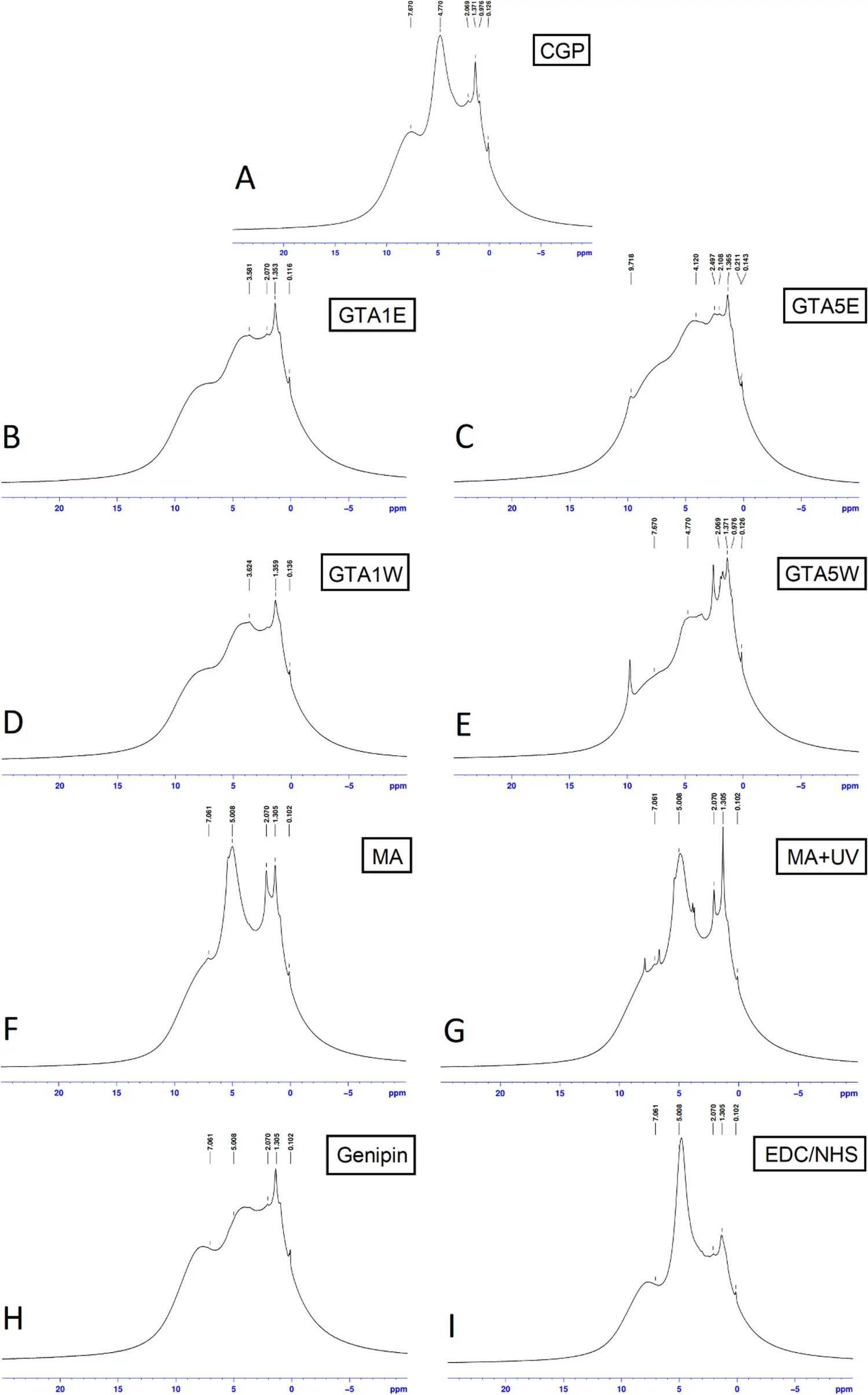
Solid-state.^1^H-NMR spectra of the samples of CGP (**A**), GTA1W (**B**), GTA5W (**C**), GTA1E (**D**), GTA5E (**E**), genipin (**F**), EDC/NHS (**G**), MA (**H**), and MA + UV (**I**)

Solid-state ^1^H-NMR spectra of the samples were obtained to examine their chemical structures after modifications (Fig. 5). The crude sample had the characteristic peaks of CGP, in which individual peaks of specific protons overlapped between 0 to 8 ppm approximately (Fig. 5A). This spectral region contained the resonances previously defined for the methyl residues of arginine at 1.72 ppm, the methylene residues of aspartic acid at 2.64 ppm, the methylene residues of lysine at 2.93 ppm, and the methylene residues of arginine at 3.14 ppm (Hu et al. 2009). Even though the individual peaks were not visible in the NMR spectrum, the accumulated effect of these peaks may be noticed between 1 and 3 ppm. The sharp peak around 4.7–4.9 ppm was assigned to the protons of water molecules within the sample structure (He et al. 1996; Aliev 2005). After GTA crosslinking, this peak was highly diminished (Fig. 5B–E), which could be attributed to the decrease in the water absorption capacity of the CGP surface followed by GTA treatment. The broadening of the proton resonances of − NH_3_^+^ groups was observed at around 7–8 ppm (Abraham et al. 2012), indicating that these groups of the samples were modified. Moreover, the crystallization peak of − NH_3_^+^ groups, formed due to the hydrogen bonds (Schmidt et al. 2006), clearly appeared at about 3.1 ppm in the GTA5E spectrum unlike the other GTA-crosslinked samples (Fig. 5E).

For the UV crosslinking, the first step (the MA incorporation into CGP) was monitored on the spectrum first (Fig. 5F). The increase in the peak sharpness at 2.07 ppm showed that the amount of − CH_2_ groups increased (Abraham et al. 2012), caused by the addition of − CH_2_-containing MA molecules to the CGP chains (Fig. 5F). After the UV crosslinking of the MA-modified CGP, the resonances observed at around 1.3 ppm were prominently sharp in the spectrum of the MA + UV group (Fig. 5G).

For the genipin-crosslinked group, the genipin structure contains aromatic moieties that would be incorporated into the CGP structure after the crosslinking process. Due to the protons of the aromatic rings in genipin, the formation of the peaks at 5 ppm (for the protons close to the OH groups) and 7 ppm (for the other protons) in the spectrum of the genipin-crosslinked group was observed (Schmidt et al. 2006) (Fig. 5H).

For the EDC/NHS-crosslinked groups, a specific sharp peak appeared at 5 ppm due to the − CH resonance (Abraham et al. 2012), which might be related to the probable increase in water affinity (Fig. 5I).

### SEM

**Fig. 6:**
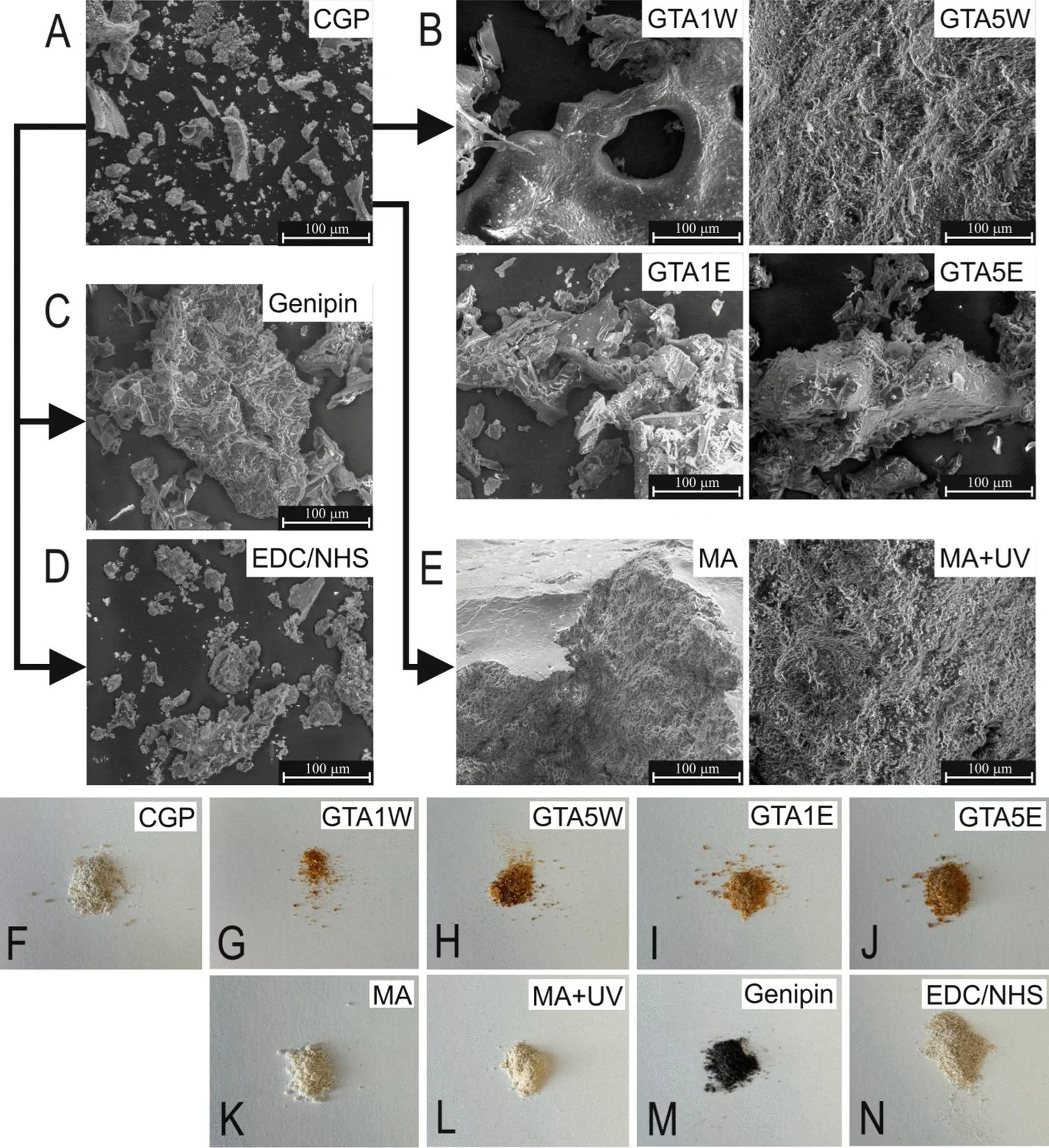
SEM images of the CGP samples before (**A**) and after the treatments by GTA (**B**), genipin (**C**), EDC/NHS (**D**), and 2-step MA + UV (**E**) (Scale bars: 100 µm). Macroscopic images of CGP (**F**), GTA1W (**G**), GTA5W (**H**), GTA1E (**I**), GTA5E (**J**), MA (**K**), MA + UV (**L**), genipin (**M**), EDC/NHS (**N**)

SEM examinations were carried out to monitor the morphological alterations in the CGP structure after the modifications (Fig. 6). The color of CGP changed from white-yellow (Fig. 6F) to orange-yellow as a result of the GTA crosslinking processes independent of the GTA concentration (Fig. 6G–J). The particle size of CGP increased after crosslinking with GTA exposure (Fig. 6B). The GTA5W group was observed in the form of an aggregated bulk with larger particle sizes compared to the GTA1W group, meaning that the higher amount of GTA resulted in a higher degree of crosslinking. The macroscopic appearance of GTA5W also confirmed the results of SEM examinations, exhibiting more or less relatively larger particles visible to the eye (Fig. 6H). However, the CGP particles did not significantly change in morphology between GTA1E and GTA5E groups in the SEM micrographs. This finding might be related to the fact that ethanol may cause conformational changes in the CGP structure (Hansen et al. 2021), which in turn, influenced the efficacy of crosslinking. Similarly, crosslinking with genipin led to the formation of larger particles (Fig. 6C), meaning that crosslinking might be achieved to a certain extent with the use of genipin as the crosslinker. On the other hand, EDC/NHS did not cause remarkable morphological changes, although some of the particles became larger in comparison to the crude form (Fig. 6D). The color of CGP converted from white-yellow (Fig. 6F) to dark blue after genipin crosslinking (Fig. 6M); however, the color of the samples remained similar after EDC/NHS crosslinking (Fig. 6N). In the last group of modifications, CGP powders were observed in a coagulated form after methacrylation and maintained similar bulky morphology after UV irradiation (Fig. 6E). The color of methacrylated and UV irradiated samples remained white-yellow (Fig. 6K, L). Overall, the SEM results displayed differences in particle size and texture of the CGP particles. At the macroscopic scale, CGP powder appearance regarding particle size changed slightly containing bulkier particulates that could be seen by the eye, although the particle size differences formed after crosslinking were not remarkable but still existed. In other words, a single solid structure was not formed by crosslinking, and the material was still in powder form. Nevertheless, a degree of morphological alterations after the treatments were observed both in microscopic and macroscopic images. Moreover, the color change could be considered another sign of crosslinking for some of the samples mentioned above.

## Cell culture studies

**Fig. 7:**
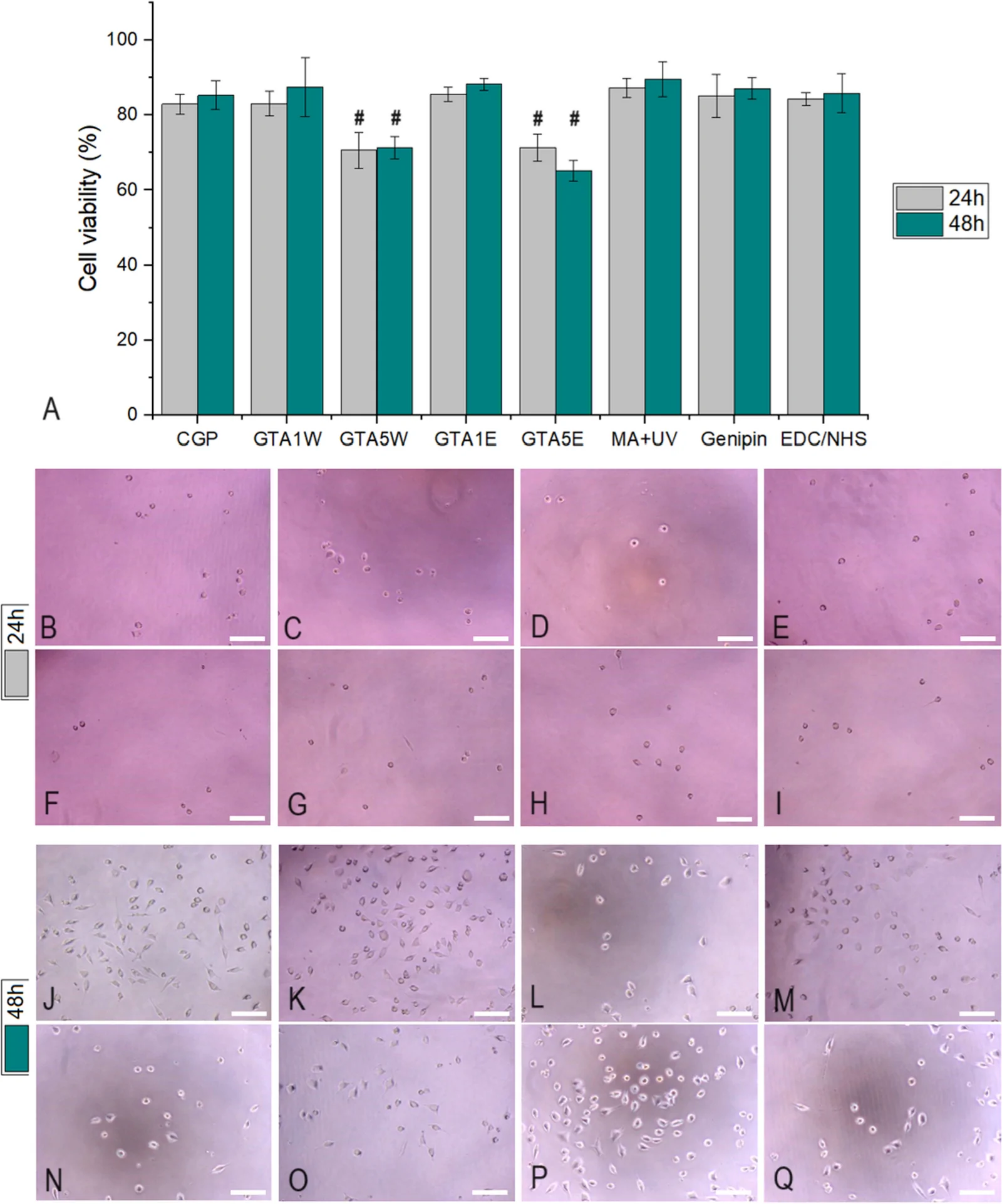
L929 cell viability results of the CGP groups treated with various crosslinkers and the uncrosslinked control after 24 and 48 h of incubation (**A**). The number sign (#) denotes the statistically lowest groups in its corresponding period. Optical microscopy images of CGP (**B**), GTA1W (**C**), GTA5W (**D**), GTA1E (**E**), GTA5E (**F**), MA + UV (**G**), genipin (**H**), EDC/NHS (**I**) after 24 h of incubation; CGP (**J**), GTA1W (**K**), GTA5W (**L**), GTA1E (**M**), GTA5E (**N**), MA + UV (**O**), genipin (**P**), EDC/NHS (**Q**) after 48 h of incubation. Scale bars: 50 µm

Cell viability studies of the CGP samples were conducted after 24 h and 48 h of incubation using the L929 cell line, and the results are shown in Fig. 7A. The cytotoxic potential of 5% of GTA-crosslinked CGP samples was noticed after 24 and 48 h, while the other samples showed statistically higher cell viability (ranging between 82 and 89%) than these two groups (*i.e.*, GTA5W and GTA5E). In the optical microscopy images, the number of cells was less for all the groups at 24 h (Fig. 7B–I). After 48 h of incubation, the lower cell density of the GTA5W and GTA5E groups became more visible compared to the rest of the groups (Fig. 7J–Q).

**Fig. 8:**
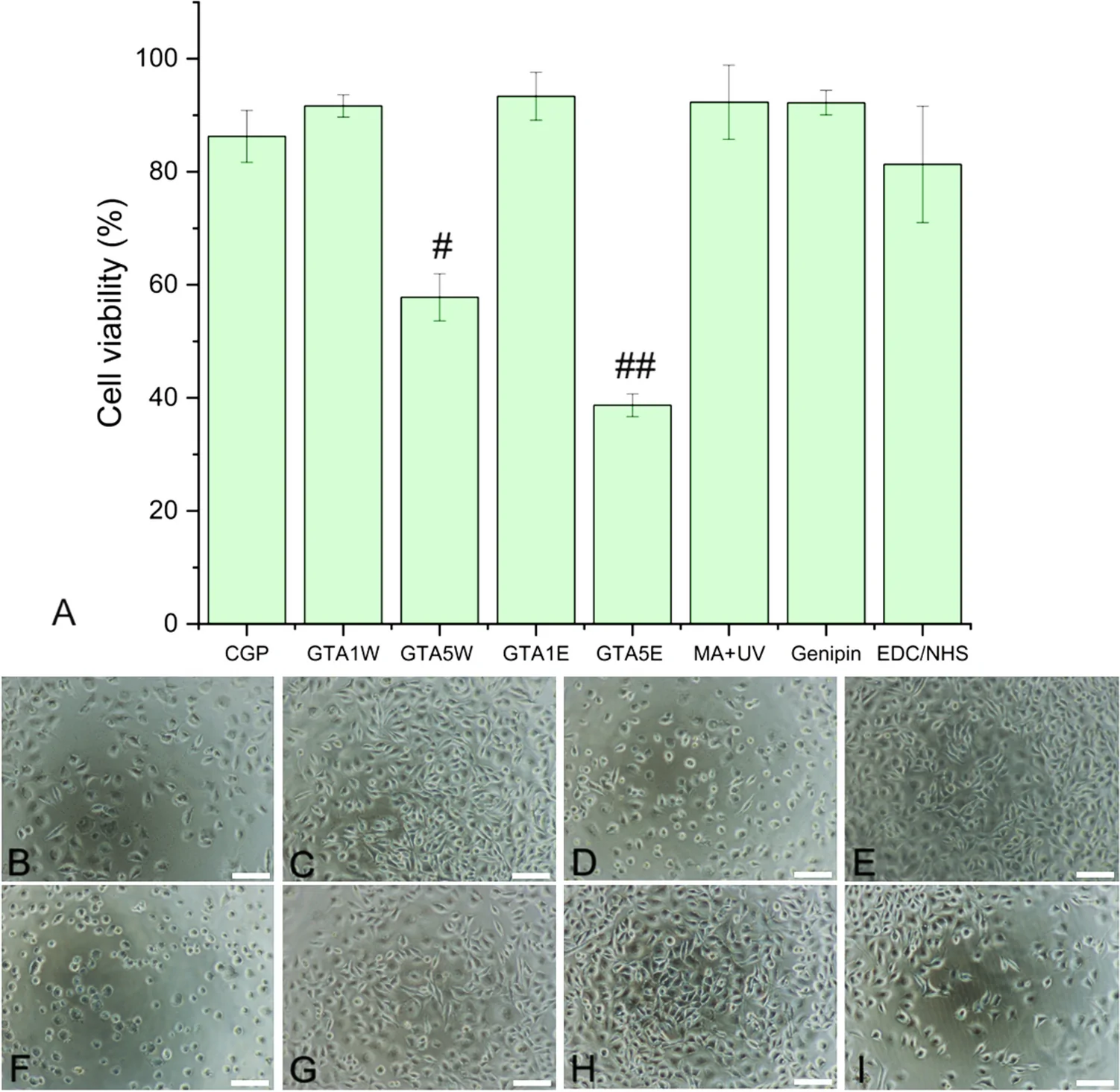
L929 cell viability results of the CGP groups treated with various crosslinkers and the uncrosslinked control after 7 days of incubation (**A**). Single-number sign (#) denotes the group statistically different than the rest. The double number sign (##) denotes statistically the lowest group. Optical microscopy images of CGP (**B**), GTA1W (**C**), GTA5W (**D**), GTA1E (**E**), GTA5E (**F**), MA + UV (**G**), genipin (**H**), EDC/NHS (**I**). Scale bars: 50 µm

Cell viability tests proceeded up to 7 days of incubation to observe the longer-term effect of CGP samples on L929 cells (Fig. 8A). The cell viability levels of all groups were above 80% except for the CGP groups crosslinked with the higher GTA concentration in water or ethanol (*i.e.*, GTA5W and GTA5E). For the CGP samples in the current study, the cell viability reached ⁓92% and ⁓93% in the GTA1W and GTA1E groups, respectively. Thus, it revealed that 1% of GTA could be considered non-cytotoxic. The concentration of this crosslinker increased up to 5% drastically reducing the cell viability (⁓58% and ⁓39% for GTA5W and GTA5E, respectively). The differences among the cell densities of the groups confirmed the cell viability results (Fig. 8B–I). For GTA5W and GTA5E groups, not only the cell density but also the cell morphology changed and became rounder, not spread (Fig. 8D, F), showing the cells were not quite healthy.

## Discussion

CGP is considered a promising biomaterial owing to its natural composition and possible non-toxic byproducts (*i.e.*, amino acids) upon biodegradation in the human body in tissue engineering applications. Therefore, initial attempts to develop novel scaffolds using CGP were presented in this study. Engineering CGP by applying chemical crosslinking (GTA, genipin, and EDC/NHS), physical crosslinking (photocrosslinking by UV light), and enzymatic (MAO) crosslinking methods was aimed to investigate their potential for the formation of stable structures. Herein, CGP was crosslinked by using several methods and characterized by analytic techniques for the first time to constitute bulky architectures from the CGP powder to be used as scaffolds.

The first presented method is crosslinking with GTA (Adamiak and Sionkowska 2020; Alavarse et al. 2022), which is one of the most commonly used chemical crosslinkers (Fig. 1). Its binding affinity with amine, imidazole, and thiol groups is high, and the procedure is simple and effective. For protein crosslinking, lysine amine groups, imidazole of histidine, and guanidyl groups of arginine take part in the crosslinking reaction (Adamiak and Sionkowska 2020). This suggests that both the abundant arginine and also the less abundant lysine of CGP could react with aldehyde groups of GTA. The disadvantage of GTA is its toxicity to cells. After the reaction, the crosslinked product should be thoroughly washed with water or molecules that bind to unreacted GTA, such as glycine (Alavarse et al. 2022). Therefore, we studied also safer methods of crosslinking that would not pose a threat to cells and would not demand additional steps of washing, such as crosslinking with genipin (Wang et al. 2019; Alavarse et al. 2022). Genipin is 10,000 times less toxic than GTA, biocompatible, biodegradable, and was used before to form stable crosslinked materials (Yu et al. 2021). In genipin crosslinking, lysine and arginine of CGP can take part in the reaction (Fig. 1). EDC/NHS crosslinking allows the production of crosslinked molecules without any additional linker, by direct covalent binding between the molecules (Fig. 1). EDC/NHS is used to chemically activate molecules (Adamiak and Sionkowska 2020; Alavarse et al. 2022). In the current study and the case of CGP, it was deduced that the covalent bond is formed between the carboxylic group of arginine/lysine and the guanidine/amine group of arginine/lysine of the second CGP molecule. The materials crosslinked by the EDC/NHS method are biocompatible and non-toxic; however, they often have poorer mechanical properties than those crosslinked by GTA (Alavarse et al. 2022).

The enzymatic method with MAO was proposed due to the known mechanism of protein crosslinking in collagen by lysyl oxidases from the MAO family (Fig. 1) (Li et al. 2018; Kong et al. 2021). Unfortunately, the amount of the product was not enough to perform all the analyses. Despite being safe and environmentally friendly, the method seems to be economically unfavorable in comparison to the other presented methods; thus, further investigations were stopped.

DSC analysis allowed us to observe the changes in the physical properties of the tested samples. When CGP was compared to the GTA-crosslinked CGP groups, the DSC curve of the crude CGP did not exhibit a significant change between 100 and 200 °C using the heat flow. This finding demonstrated the highly amorphous structure of the crude CGP (Abd El-Kader et al. 2010) (Fig. 3). This result was expected since the crosslinked samples were supposed to be restricted with a lowered number of amorphous regions due to the crosslinking. Crude CGP had an endothermic peak at 89 °C. All crosslinked samples exhibited similar endothermic peaks, from 80 to 91 °C. The lowest value was obtained for CGP crosslinked with 1% GTA in water (GTA1W) (80 °C). For GTA crosslinked samples in ethanol (GTA1E and GTA5E), the endothermal peak was observed closer to that of CGP (86–89 °C). It was previously reported that two phenomena influenced the denaturation peaks of bovine hide collagens; (i) after GTA crosslinking of the proteins, the peak shifted from lower to upper temperatures since the chain mobility declined and thus, the thermal stability increased, and (ii) the more the samples were moisturized, the more the peak decreased since the mobility was enhanced by the moisture content (Schroepfer and Meyer 2017). In the moisture aspect, CGP, GTA5W, GTA1E, and GTA5E groups might possess a similar level of dryness. However, GTA1W with the lower peak value probably absorbed more water molecules compared to the others. On the other hand, it was also demonstrated that, besides the increase in the denaturation peak, the enthalpy of the peak became remarkably smaller when the crosslinking efficiency increased (Bigi et al. 2001; Schroepfer and Meyer 2017). In this regard, the GTA5W group exhibited the lowest enthalpy degree that corresponded to the higher level of crosslinking compared to the other GTA-crosslinked groups.

For the CGP crosslinked with genipin and EDC/NHS, the peak characteristics were similar to each other with more exothermic tails at the end than that of the crude CGP group (Fig. 3). As mentioned above, such greater enthalpy levels (compared to the control) might be attributed to some losses in the amorphous regions of CGP after crosslinking of the peptide chains, consistent with the report on genipin-crosslinked gelatin samples (Bigi et al. 2002). The peaks of genipin and EDC/NHS-crosslinked groups were observed at 87 °C and 85 °C, respectively which were slightly lower than that of CGP (89 °C). This result might indicate the increase in the water-holding capacity of CGP after crosslinking with genipin or EDC/NHS due to the hydrophilicity of these crosslinkers (Huang et al. 2018; Mak and Leung 2019).

For the methacrylated samples, the thermal stability decreased considerably (endothermic peak at 59 °C), while after crosslinking with UV, the stability enhanced (peak at 79 °C) (Fig. 3). This result was also consistent with the findings of the TGA analysis. The peak shift caused by the first modification of CGP (*i.e.*, the addition of methacrylate groups) must have been related to significant changes in chain interactions within CGP—(i) tightly packed peptide chains might come apart from each other by the incorporation of the methacrylate side groups, and (ii) positively charged guanidine groups of arginine might be replaced by -CH_2_ and -CH_3_, which decreases the overall charge of the polymer. Enhancement of thermal stability after crosslinking is often stated in literature (Marin et al. 1964; Jia et al. 2019). The second shift of the endothermic peak (from 59 to 79 °C) pointed out the UV crosslinking of the methacrylate samples.

In the literature, it was reported that DSC curves of soluble and insoluble CGP had no endothermic peaks and lacked Tg due to the limited mobility of polypeptide chains (Khlystov et al. 2017), unlike ours. These findings might imply that CGP properties could change drastically depending on the source or batch, which should be characterized before any application. Consequently, herein, it was hypothesized that the mobility of the chains of CGP was already heavily restricted due to interaction between its chains; thus, the differences after crosslinking would not be significantly distinguished in DSC analyses.

Thermal degradation of the samples was studied by TGA analysis (Figs. 4 and S2, Table 1). The temperature at the onset of decomposition was slightly increased for crosslinked CGP. It was concluded that the crosslinking process disrupted the highly aggregated and tightly packed sub-regions of CGP making it less thermally stable. The largest mass loss was observed for all samples in the temperature range of 280–600 °C (from 20% for crude CGP to 55% for MA + UV), and at this stage, the rate of degradation most significantly varied between the materials (Table 1).

Solid-state ^1^H-NMR spectra of the samples were obtained to prove the success of the crosslinking reaction on the molecular level (Fig. 5). The broad peak observed between 1 and 3 ppm was an indication of the characteristic groups belonging to the crude CGP (Fig. 5A). The decrease in the peak corresponding to the water molecules (4.7–4.9 ppm) within CGP was an expected result after GTA crosslinking (Fig. 5B–E). It was reported that the water absorption capacity tended to get lowered after crosslinking due to the hydrophobization effect of GTA (Beppu et al. 2007). Another indication of modification is the broadening of the proton resonances of − NH_3_^+^ groups observed at around 7–8 ppm (Abraham et al. 2012) (Fig. 5E). GTAE5 groups specifically exhibited a crystallization effect compared to the other groups. It was caused by the well-known crystallization effect of ethanol on proteins or polypeptide structures (Hansen et al. 2021). The resonances that appeared near 0 ppm were mostly associated with a small number of impurities for all the samples (He et al. 1996).

The origin of the sharp peaks may be diverse: unreacted crosslinking reagents, solvent used during the reactions, degradation product enclosed in the network, etc. The changes visible in the NMR spectra between crude CGP and the crosslinked CGP proved the success of the reactions.

SEM analyses showed that the crude CGP was converted more efficiently into bulky structures after GTA crosslinking (GTA5W) and photocrosslinking (MA + UV) compared to the others (Fig. 6A–E). The other crosslinked groups exhibited relatively lower degrees of morphological changes when compared to these GTA5W and MA + UV groups. In the concept of scaffolding for tissue engineering applications, the porosity of the samples would be an issue since porous scaffolds are required for maintaining cell migration into them. However, the porosity of CGP samples can be improved by using common methods such as salt-leaching. Furthermore, different materials may be utilized to hybridize CGP scaffolds, and then the lyophilization applied for drying the samples can be useful to generate pores within the bulky structures. Consequently, these first characterizations of crosslinked CGP can be beneficial for future studies that will be carried out to investigate the properties of candidate CGP scaffolds or CGP-containing scaffolds. The modifications can be altered to increase the crosslinking efficacy and/or add new features to the CGP biomaterials concerning specific problem–solution cases in the field of tissue engineering. For instance, the biodegradation rate of potential CGP scaffolds can be tailored by playing with the crosslinking methods, crosslinker concentrations, and experimental conditions, as well as release rate and mechanisms of potential drug-loaded CGP scaffolds or particles can be optimized. Considering that these CGP samples were not soluble in water before or after any of these crosslinking treatments, the modification technique should be chosen accordingly during the optimizations.

The cell viability levels of all groups were above 80% except for the CGP groups crosslinked with the higher GTA concentration in water or ethanol (*i.e.*, GTA5W and GTA5E). It was reported that the cytotoxicity level of GTA changed greatly depending on the study (Reddy et al. 2015). Even though GTA is a highly effective crosslinker that could improve the stability of the final product by bonds mostly formed with primary amine groups (Frick et al. 2018; Anastassacos et al. 2020), its cytotoxicity range must be determined for each biomaterial design. Herein, the current study revealed that 1% of GTA for CGP crosslinking could be considered non-cytotoxic. The concentration of this crosslinker should not be increased up to 5% because it drastically reduced the cell viability (⁓58% and ⁓39% for GTA5W and GTA5E, respectively). The risks of the UV crosslinking process for cell viability could be listed as phototoxicity (*e.g.*, UV light wavelength and UV exposure time) and radical toxicity (*e.g.*, photoinitiator concentration and free radicals generated during the process) (Lim et al. 2020). In our case, phototoxicity would not pose any threat since the samples were exposed to UV light before application and would come in contact with the cells in the absence of UV. However, the radical toxicity might affect the cell viability, and it was investigated. According to the results, the cells exhibited approximately 92% viability in the UV-crosslinked CGP group, meaning that they were non-cytotoxic. As a natural crosslinker, genipin is well-known for its non-toxic features (Filová et al. 2020). Consistently, the cell viability of the genipin-crosslinked CGP group was measured as around 92%, confirming its biocompatibility potential in the human body. Lastly, the EDC/NHS group was tested, and the cells maintained ⁓81% viability. This group was also classified as a non-toxic material according to the ISO standards (*i.e.*, cell viability above 70% (Lopes et al. 2016)). The microscope images were aligned with the cell viability results (Figs. 7J–Q and 8B–I). In the high-percent GTA groups, not only the cell densities were significantly lower but also the cells had round morphology (Fig. 8D and F). In the GTA5E group, the shape of the cells became even more rounder, meaning that they were unhealthy and could not spread well, which is consistent with the cell viability results.

Crosslinking with GTA and the solvent type used during the procedure resulted in chemical and morphological changes in the CGP structure. If water and ethanol are compared as solvents, ethanol probably caused conformational changes in the structure of CGP samples and resulted in a decreased efficiency of crosslinking. Crosslinking with genipin or EDC/NHS exhibited similar influences on CGP. Physical crosslinking of CGP by UV light was carried out after the incorporation of methacrylate side groups into the CGP chains. This modification caused a decrease in the thermal stability of CGP, which was followed by the enhancement of the stability after UV exposure. The yield of the enzymatic crosslinking by using monoamine oxidase was found insufficient to be suggested for large-scale production. In vitro studies demonstrated that CGP crosslinked with low-concentration GTA, UV light, genipin, and EDC/NHS was cytocompatible. In light of the current investigations, CGP might be regarded as a convenient alternative for scaffolding purposes in the composite form after hybridization with other biomaterials and optimization of the properties concerning the requirements of the specific tissue engineering application for the targeted defect site. The properties of the polymers that would be combined with CGP samples should be also taken into account since they would change the overall characteristics of the final scaffolds. In this regard, the results represented only for CGP may not be appropriate and adequate. Herewith, even though the potential of CGP was examined partially using chemical, physical, and biological features, further in vitro and in vivo testing is necessary to fully assess the feasibility of CGP-containing scaffolds for tissue engineering and related biomedical applications.

## Supplementary Information

Below is the link to the electronic supplementary material.

Supplementary file1 (PDF 450 KB)

## Data Availability

All data generated or analyzed during this study are included in this published article and its supplementary information file.
