# Supplementary material for: Cyanophycin modifications for applications in tissue scaffolding
Source: Appl Microbiol Biotechnol. 2024 Mar 15;108(1):264. doi: 10.1007/s00253-024-13088-4 (PMC10943155; doi:10.1007/s00253-024-13088-4)
Supplement: Supplementary file 1 — Supplementary file1 (PDF 450 KB) [file 253_2024_13088_MOESM1_ESM.pdf]

Supporting Information for publication

Cyanophycin modifications for applications in tissue scaffolding

Natalia Kwiatos[a#], Deniz Atila[a#], Michał Puchalski[b], Vignesh Kumaravel[a], Alexander Steinbüchel[a]*

[a]International Centre for Research on Innovative Biobased Materials – International Research Agenda (ICRI-BioM), Lodz University of Technology, Stefanowskiego 2/22. Łódź, Poland, natalia.kwiatos@p.lodz.pl

[b] Institute of Material Science of Textiles and Polymer Composite, Lodz University of Technology, Żeromskiego 116, Łódź, Poland

[#] These authors contributed equally and are first authors

KEYWORDS: Cyanophycin; Multi-l-arginyl-poly-l-aspartate; crosslinking; Glutaraldehyde; Genipin; EDC/NHS;

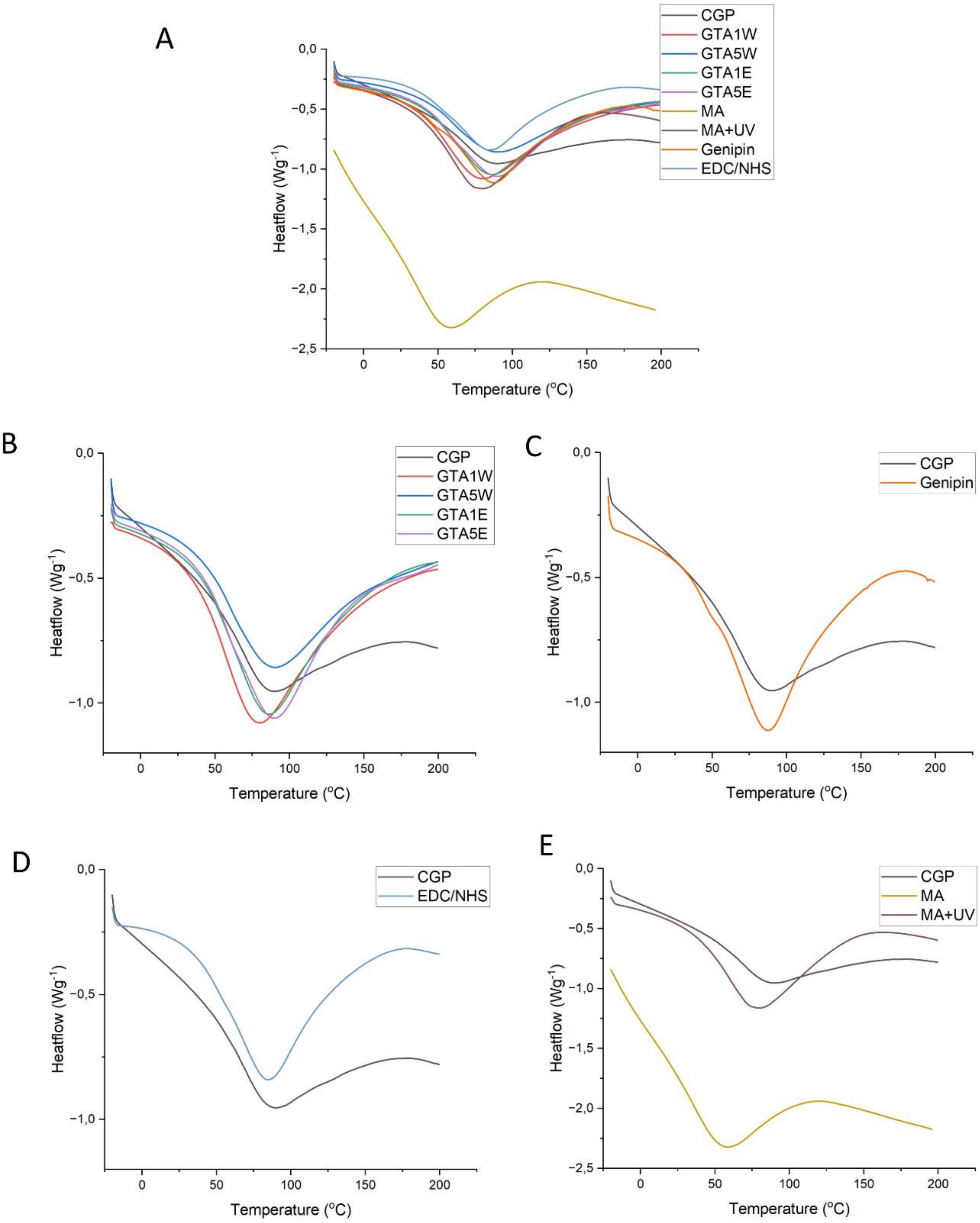

Figure S1 DSC thermograms of the samples after all modifications (A) and their split versions for GTA (B), genipin (C), EDC/NHS (D), and MA+UV (E).

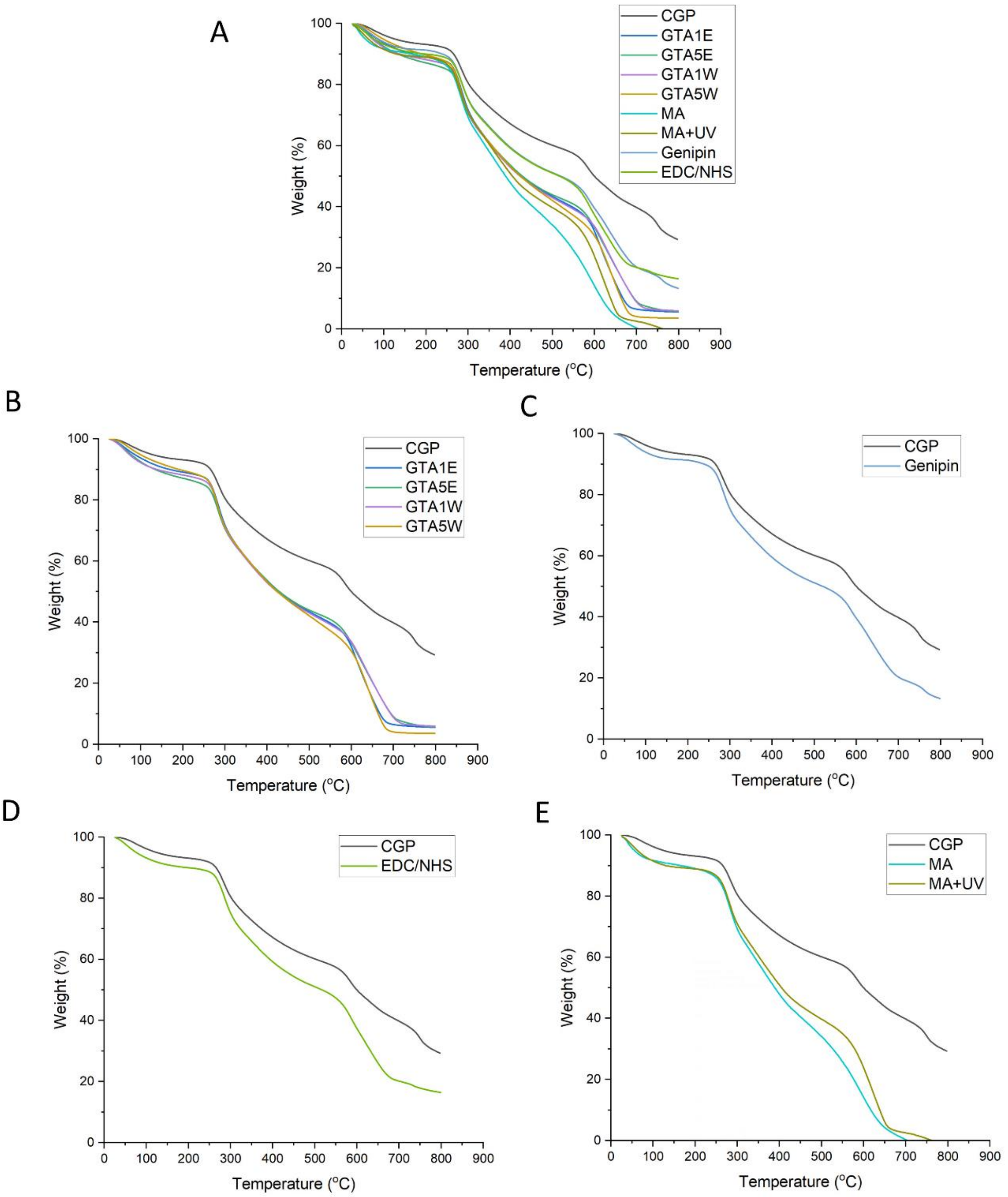

Figure S2 TGA curves of the samples after all modifications (A) and their split versions for GTA (B), genipin (C), EDC/NHS (D), and MA+UV (E).
